# Hemoglobin as a possible biochemical index of hypertension-induced vascular damage

**DOI:** 10.1186/s40101-016-0085-7

**Published:** 2016-01-28

**Authors:** Yuji Shimizu, Koichiro Kadota, Mio Nakazato, Yuko Noguchi, Jun Koyamatsu, Hirotomo Yamanashi, Mako Nagayoshi, Shuichi Nagata, Kazuhiko Arima, Takahiro Maeda

**Affiliations:** Nagasaki University Graduate School of Biomedical Science, Nagasaki, Japan

## Abstract

**Background:**

We previously reported on the positive association of hemoglobin with hypertension and atherosclerosis. On the other hand, hepatocyte growth factor (HGF) has been evaluated as a possible biochemical index of hypertension-induced vascular damage. However, no studies have reported on a correlation between hemoglobin and HGF accounting for hypertension status.

**Methods:**

A cross-sectional study of 1108 subjects (392 men and 716 women, 40–93 years old) who were undergoing a general checkup in 2014 was conducted.

**Results:**

Multiple linear regression analysis adjustment for known cardiovascular risk factors showed no significant correlation between hemoglobin and HGF in non-hypertensive subjects, but a significant positive correlation in hypertensive subjects; *β* (parameter estimate) = 0.3 (*p* = 0.975) for non-hypertensive men, *β* = 0.4 (*p* = 0.925) for non-hypertensive women, *β* = 32.7 (*p* < 0.001) for hypertensive men, and *β* = 18.7 (*p* = 0.002) for hypertensive women.

**Conclusion:**

We found a significant positive correlation between hemoglobin and HGF among hypertensive men and women. Like HGF, hemoglobin may be a useful indicator to evaluate hypertension-induced vascular damage. Since hemoglobin can easily be measured, these results support hemoglobin as an efficient tool to evaluate vascular damage induced by hypertension in daily medical practice.

## Background

We reported independent positive associations of hemoglobin level with hypertension [1] and atherosclerosis [2] in both men and women. On the other hand, endothelial dysfunction may contribute to the increased vascular tone seen with hypertension [3]. Hemoglobin might therefore serve as an indicator of vascular damage induced by hypertension. Hepatocyte growth factor (HGF), which is known as an angiogenic growth factor [4, 5], plays an important role in endothelial maintenance (vascular maintenance) and endothelial repair (vascular repair). HGF prevents apoptosis of endothelial cells [6, 7] and suppresses xanthin oxidase activation [8] induced by hypoxia/reoxygenation. Furthermore, HGF promotes endothelial cell differentiation and increases endothelial progenitor cell migration and proliferation [9]. However, other studies have reported HGF as a possible biochemical index of vascular damage due to hypertension [4, 10–14]. Another study reported a significant correlation between increased HGF concentration and carotid atherosclerosis [15]. These studies indicate that high plasma concentration of HGF indicates the presence of aggressive vascular remodeling, which results in atherosclerosis. Since HGF is constitutively produced by human bone marrow and indirectly promotes the growth of undifferentiated hematopoietic cells and erythroid progenitor cells [16], HGF level should correlate with hemoglobin, particularly when the bone marrow becomes activated by hypertension. Hemoglobin is an easily measured parameter; therefore, if it can substitute for HGF as a possible biochemical index of vascular damage due to hypertension [4, 10–14], it could serve as an efficient tool for blood pressure control in daily medical practice. Since the prevalence of hypertension might play an important role as a vascular impairment factor, the correlation between hemoglobin and HGF should account for hypertension status. However, no studies have reported a correlation between hemoglobin and HGF accounting for hypertension status. We therefore hypothesized that non-hypertensive men and women would show no significant correlation between hemoglobin and HGF, whereas a significant correlation would be found in subjects with hypertension.

To investigate these possible correlations, we conducted a cross-sectional study of 1108 subjects (392 men and 716 women, 40–93 years) who were undergoing general heath checkups in 2014.

## Subjects and methods

### Subjects

The study was conducted during a medical screening program for members of the general population aged 40–99 years who were living in Goto city, Nagasaki Prefecture, Japan. After obtaining informed consent, 1555 Japanese subjects (536 men and 1019 women) were enrolled. Subjects without habitual status (drinking, smoking) data (1 male, 1 female) and/or without blood sample data (143 males, 301 females) were excluded. To avoid the influence of abnormal HGF values, one woman with extremely high HGF (4825 pg/mL) was excluded, leaving a total of 1108 subjects (392 men, 716 women) participating in the study. This study was approved by the Ethics Committee for Human Use of Nagasaki University (project registration number 14051404).

### Data collection and laboratory measurements

Systolic and diastolic blood pressures at rest in a sitting position were recorded using a blood pressure measuring device (HEM-907; Omron, Kyoto, Japan) by trained technicians.

Height and weight in bare feet and light clothing were measured, and body mass index (BMI) was calculated as weight (kg)/(height (m))^2^. Trained interviewers obtained information on smoking and drinking status. Fasting blood samples were collected in an EDTA-2K tube and a siliconized tube. Samples from the siliconized tube were centrifuged after blood coagulation, and the separated serum was collected. Samples from the EDTA-2K tube were used to measure hemoglobin using the sodium lauryl surfate (SLS)-hemoglobin method at SRL, Inc. (Tokyo, Japan). This method is recommended by the International Committee for Standardization in hematology. Serum triglyceride (TG), serum high-density lipoprotein (HDL) cholesterol, serum low-density lipoprotein (LDL) cholesterol, serum aspartate aminotransferase (AST), serum γ-glutamyltranspeptidase (γ-GTP), hemoglobin (Hb)A_1C_, and serum creatinine were measured using standard laboratory procedures at SRL, Inc. (Tokyo, Japan). To measure HGF, serum samples were diluted fourfold with specific Bio-Plex sample diluents. HGF concentration was determined using a fluorescent bead-based immunosorbent assay on a suspension array. Glomerular filtration rate (GFR) was estimated using an established method with three variations that were recently proposed by a working group of the Japanese Chronic Kidney Disease Initiative [17]. According to this adaptation, GFR (mL/min/1.73 m^2^) = 194 × (serum creatinine (enzyme method))^−1.094^ × (age)^−0.287^ × (0.739 for women). Hypertension was defined as a systolic blood pressure ≥140 mmHg and/or a diastolic blood pressure ≥90 mmHg.

### Statistical analysis

Sex-specific models were conducted. Difference in mean ± standard deviation (SD) values, the prevalence of potential confounding factors, and *p* values by hypertension status were calculated. Simple correlation coefficients of HGF and other variables stratified by hypertension were calculated. Simple and multiple linear regression analyses stratified by hypertension were performed to evaluate the correlation between hemoglobin and HGF. Probability values less than 0.05 were considered to indicate statistical significance. All statistical analyses were performed with the SAS system for Windows (version 9.3; SAS Inc., Cary, NC).

## Results

Among the study population, 512 individuals (198 men and 314 women) were recognized as having hypertension. Sex-specific characteristics of the study population by hypertension status are shown in Table 1. Both men and women subjects with hypertension showed significantly higher levels of hemoglobin and BMI than subjects without hypertension. The mean ± SD values of hemoglobin and BMI for non-hypertensive and hypertensive men were 14.5 ± 1.3 g/dL, 22.7 ± 2.7 kg/m^2^ and 14.7 ± 1.2 g/dL, 23.7 ± 3.1 kg/m^2^ (*p* = 0.025 and *p* < 0.001), respectively; and the corresponding values for non-hypertensive and hypertensive women were 12.9 ± 1.1 g/dL, 22.3 ± 3.5 kg/m^2^ and 13.2 ± 1.2 g/dL, 23.1 ± 3.3 kg/m^2^ (*p* = 0.003 and *p* = 0.004), respectively.

**Table 1:** Sex-specific characteristics of the study population by hypertension status

	Men			Women		
	Hypertension			Hypertension		
	(−)	(+)	*p*	(−)	(+)	*p*
No. of cases	194	198		402	314	
Age	69.4 ± 9.4	69.9 ± 9.1	0.612	67.3 ± 9.7	72.9 ± 8.9	<0.001
Hepatocyte growth factor (HGF), pg/mL	279.7 ± 126.8	284.6 ± 150.9	0.730	239.6 ± 88.7	256.1 ± 119.8	0.035
Hemoglobin (Hb), g/dL	14.5 ± 1.3	14.7 ± 1.2	0.025	12.9 ± 1.1	13.2 ± 1.2	0.003
Systolic blood pressure, mmHg	123 ± 10	151 ± 13	<0.001	124 ± 10	153 ± 14	<0.001
Diastolic blood pressure, mmHg	76 ± 9	91 ± 10	<0.001	74 ± 9	86 ± 11	<0.001
Body mass index (BMI), kg/m^2^	22.7 ± 2.7	23.7 ± 3.1	<0.001	22.3 ± 3.5	23.1 ± 3.3	0.004
Curent drinker, %	53.1	63.6	0.034	17.7	14.0	0.188
Curent smoker, %	16.5	18.2	0.660	3.7	1.6	0.085
Serum HDL cholesterol, mg/dL	56 ± 15	57 ± 14	0.588	63 ± 14	61 ± 14	0.049
Serum LDL cholesterol, mg/dL	109 ± 28	111 ± 29	0.517	120 ± 28	124 ± 29	0.052
Serum triglyceride (TG), mg/dL	108 ± 72	122 ± 111	0.144	99 ± 53	114 ± 62	<0.001
Serum aspartate transaminase (AST), IU/L	25 ± 9	26 ± 11	0.096	22 ± 6	23 ± 7	0.127
Serum γ-glutamyltranspeptidase (γ-GTP), IU/L	34 ± 28	51 ± 79	0.004	23 ± 19	21 ± 14	0.073
Hemoglobin A1c (HbA1c), %	5.6 ± 0.5	5.8 ± 0.9	0.109	5.6 ± 0.4	5.7 ± 0.5	0.137
Glomerular filtration rate (GFR), mL/min/1.73 m^2^	69.1 ± 15.2	69.4 ± 13.8	0.847	68.8 ± 14.1	66.5 ± 13.6	0.026

**Fig. 1:**
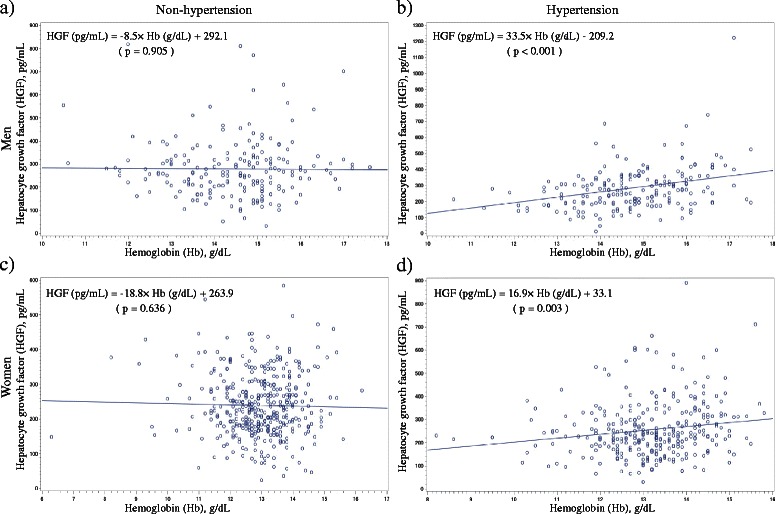
Simple linear regression analysis of hepatocyte growth factor (HGF) and with hemoglobin (Hb) in **a** non-hypertensive men, **b** hypertensive men, **c** non-hypertensive women, and **d** hypertensive women

The simple correlation coefficients of HGF and other variables stratified by hypertension status are shown in Table 2. In non-hypertensive men and women, no significant correlation between hemoglobin and HGF was seen, whereas a significant positive correlation was seen among subjects with hypertension. This was also seen from simple linear regression analysis (Fig. 1). From multiple linear regression analysis adjustment for known cardiovascular risk factors, significant positive correlation between hemoglobin and HGF was observed in both men (*β* (parameter estimate) = 32.7, *p* < 0.001) and women (*β* = 18.7, *p* = 0.002) with hypertension (Tables 3 and 4).

**Table 2:** Sex-specific simple correlation coefficient of hepatocyte growth factor (HGF) and other variables

	Men				Women			
	Hypertension (−)		Hypertension (+)		Hypertension (−)		Hypertension (+)	
	*r*	*p*	*r*	*p*	*r*	*p*	*r*	*p*
Age	0.11	0.123	−0.16	0.020	0.15	0.003	0.10	0.091
Systolic blood pressure	0.19	0.009	−0.02	0.737	0.07	0.156	−0.02	0.720
Diastolic blood pressure	0.18	0.013	0.08	0.250	−0.06	0.269	0.03	0.589
Body mass index (BMI)	0.03	0.638	0.33	<0.001	0.16	0.001	0.13	0.027
Drinking status	−0.03	0.642	−0.01	0.936	−0.06	0.257	0.05	0.370
Smoking status	0.14	0.059	0.12	0.086	−0.004	0.937	0.06	0.301
Serum HDL cholesterol	−0.12	0.107	−0.08	0.237	−0.14	0.004	−0.08	0.140
Serum LDL cholesterol	−0.07	0.346	−0.09	0.196	−0.07	0.146	−0.10	0.081
Serum triglycerides (TG)	0.04	0.618	0.09	0.187	0.01	0.845	0.04	0.510
Serum aspartate transaminase (AST)	0.01	0.888	0.25	<0.001	0.02	0.647	0.11	0.047
Serum γ-glutamyltranspeptidase (γ-GTP)	0.03	0.705	0.26	<0.001	−0.01	0.834	0.11	0.043
Hemoglobin A1c (HbA1c)	0.11	0.117	0.05	0.507	0.11	0.031	0.17	0.002
Glomerular filtration rate (GFR)	−0.04	0.542	0.09	0.223	−0.09	0.074	−0.01	0.869
Hemoglobin (Hb)	−0.01	0.905	0.28	<0.001	−0.02	0.636	0.17	0.003

Drinking status (never drinker, former drinker, current drinker (<23 g/week, 23–45 g/week, 46–68 g/week, ≥69 g/week)), smoking status (never smoker, former smoker, current smoker)

**Table 3:** Multiple linear regression analysis of hepatocyte growth factor (HGF) with relevant factors adjusted for confounding factors among men

	Hypertension (−)			Hypertension (+)		
	*β*	95 % CI	*p*	*β*	95 % CI	*p*
No. of participants		194			198	
Age	1.3	(−0.9, 3.5)	0.255	−0.3	(−3.0, 2.4)	0.815
Systolic blood pressure	0.6	(−1.9, 3.2)	0.620	0.1	(−1.5, 1.7)	0.923
Diastolic blood pressure	2.4	(−0.5, 5.3)	0.110	−1.1	(−3.5, 1.4)	0.396
Body mass index (BMI)	−1.7	(−9.5, 6.1)	0.668	11.2	(3.9, 18.5)	0.003
Drinking status	−4.0	(−13.8, 5.7)	0.413	−5.5	(−14.8, 3.9)	0.250
Smoking status	25.8	(−3.2, 54.9)	0.081	11.3	(−20.5, 43.2)	0.484
Serum HDL cholesterol	−0.9	(−2.4, 0.7)	0.276	−0.4	(−1.9, 1.2)	0.663
Serum LDL cholesterol	−0.4	(−1.1, 0.2)	0.195	−0.4	(−1.2, 0.3)	0.259
Serum triglycerides (TG)	−0.04	(−0.3, 0.3)	0.800	−0.2	(−0.4, 0.03)	0.091
Serum aspartate aminotransferase (AST)	−0.4	(−2.7, 2.0)	0.757	1.6	(−0.7, 3.8)	0.170
Serum γ-glutamyltranspeptidase (γ-GTP)	0.2	(−0.6, 1.0)	0.602	0.4	(0.04, 0.8)	0.029
Hemoglobin A1c (HbA1c)	14.1	(−20.8, 48.9)	0.427	6.5	(−16.8, 29.9)	0.582
Glomerular filtration rate (GFR)	0.1	(−1.2, 1.4)	0.882	0.1	(−1.4, 1.6)	0.906
Hemoglobin (Hb)	0.3	(−15.9, 16.4)	0.975	32.7	(14.4, 51.0)	<0.001

Drinking status (never drinker, former drinker, current drinker (<23 g/week, 23–45 g/week, 46–68 g/week, ≥69 g/week)), smoking status (never smoker, former smoker, current smoker)

**Table 4:** Multiple linear regression analysis of hepatocyte growth factor (HGF) with relevant factors adjusted for confounding factors among women

	Hypertension (−)			Hypertension (+)		
	*β*	95 % CI	*p*	*β*	95 % CI	*p*
No. of participants		402			314	
Age	1.1	(−0.02, 2.2)	0.055	2.6	(0.8, 4.5)	0.006
Systolic blood pressure	0.4	(−0.6, 1.5)	0.418	−0.7	(−1.7, 0.3)	0.181
Diastolic blood pressure	−0.5	(−1.7, 0.8)	0.450	0.9	(−0.6, 2.3)	0.231
Body mass index (BMI)	3.7	(1.0, 6.3)	0.007	1.5	(−2.7, 5.8)	0.482
Drinking status	−1.8	(−8.0, 4.4)	0.562	4.3	(−6.3, 15.0)	0.426
Smoking status	11.0	(−9.6, 31.6)	0.293	25.2	(−15.2, 65.5)	0.220
Serum HDL cholesterol	−0.8	(−1.5,−0.1)	0.023	−1.0	(−2.0, 0.05)	0.061
Serum LDL cholesterol	−0.2	(−0.6, 0.1)	0.129	−0.4	(−0.8, 0.1)	0.124
Serum triglycerides (TG)	−0.1	(−0.3, 0.04)	0.122	−0.1	(−0.3, 0.2)	0.598
Serum aspartate aminotransferase (AST)	−0.3	(−1.9, 1.2)	0.652	1.4	(−0.5, 3.3)	0.146
Serum γ-glutamyltranspeptidase (γ-GTP)	0.05	(−0.4, 0.5)	0.851	0.5	(−0.5, 1.5)	0.353
Hemoglobin A1c (HbA1c)	11.5	(−10.3, 33.4)	0.301	41.7	(16.6, 66.9)	0.001
Glomerular filtration rate (GFR)	−0.2	(−0.9, 0.5)	0.627	0.3	(−0.7, 1.4)	0.525
Hemoglobin (Hb)	0.4	(−7.5, 8.3)	0.925	18.7	(6.7, 30.7)	0.002

Drinking status (never drinker, former drinker, current drinker (<23 g/week, 23–45 g/week, 46–68 g/week, ≥69 g/week)), smoking status (never smoker, former smoker, current smoker)

## Discussion

A major finding of this study was a significant positive correlation between hemoglobin and HGF among hypertensive men and women subjects, but not among non-hypertensive subjects.

**Fig. 2:**
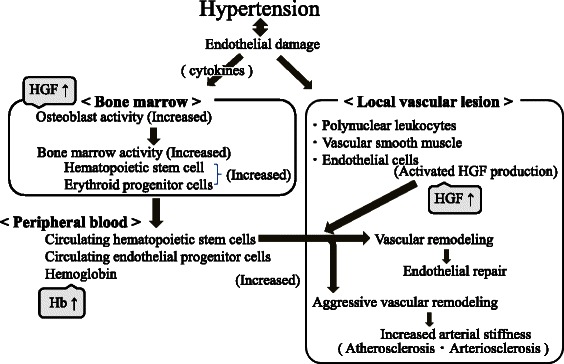
Possible mechanism of endothelial dysfunction and repair in relation to hypertension

The association between hypertension and endothelial dysfunction is bidirectional. In a state of hypertension, oxidative stress increases due to increased NADH/NADPH oxidase activation, while oxidative stress inactivates nitric monoxide (NO), which is known as an endothelial-dependent vasodilation factor, by causing injury to endothelial cells, resulting in increased vasoconstriction [18]. Hypertension is therefore maintained. In addition to this mechanism, endothelial dysfunction causes reduced NO production, and increased endothelial-dependent vasoconstriction factors such as endothelin, angiotensin II, and thromboxane are also observed in hypertension [19]. The number of growth factors in circulating blood was reported to increase when complications consisting of hypertension arose in response to vascular endothelial cell damage [20]. Additionally, HGF is reported to be produced by polynuclear leukocytes [21], vascular smooth muscle cells, endothelial cells in humans in vivo [22], and human osteoblasts in vitro [23], and unlike most known growth factors, HGF acts specifically on the endothelium and does not promote the growth of smooth muscle cells [5]. Furthermore, one study reported that the overexpression of HGF in smooth muscle cells can be beneficial for promoting endothelial cell differentiation and increasing endothelial progenitor cell migration and proliferation [9]. However, human endothelial progenitor cells (CD34-positive cells) are reported to differentiate into not only endothelial cells but also into foam cells, which are a contributing factor in the development of atherosclerosis [24]. Other studies have reported the presence of CD34-positive cells in human atherosclerotic lesions [25, 26]. Furthermore, increased HGF concentration significantly correlates with carotid atherosclerosis [15]. Therefore, even though HGF has a beneficial effect on vascular endothelial repair, subjects with a high plasma HGF concentration likely have a high risk of atherosclerosis (endothelial dysfunction). Additionally, HGF is reported to indirectly promote the growth of undifferentiated hematopoietic cells (CD34-positive cells) and erythroid progenitor cells (source of hemoglobin) [16]. Also, because osteoblasts regulate the production of hematopoietic stem cells in the bone marrow [27, 28], and HGF is reported to be produced by osteoblasts [23], high levels of plasma HGF and hemoglobin might also indicate high activity of the bone marrow. Since hematopoietic stem cells derived from the bone marrow play a major role in vascular homeostasis [29–31], and other studies have reported that hemoglobin plays an important role in the progression of atherosclerosis [32, 33], hemoglobin level significantly correlates with HGF when the bone marrow is activated by endothelial damage induced by hypertension. In our previous study, we reported that hemoglobin levels in both men and women are positively associated with hypertension [1] and atherosclerosis [2]. Other studies identified HGF as a possible biochemical index of hypertension-induced vascular damage [4, 10–14]. These studies partly support the abovementioned mechanisms. A possible mechanism underlying the observed correlation between hemoglobin and HGF in hypertensive subjects is summarized in Fig. 2.

Our findings should be interpreted with some caution. Although hemoglobin, which might act as a marker of bone marrow activity, showed a significant positive correlation with HGF, we were not able to evaluate the influence of hematopoietic stem cells. Further investigations using hematopoietic stem cell data are necessary. Finally, since this was a cross-sectional study, no causal relationships were able to be established.

## Conclusion

In conclusion, an independent positive correlation between hemoglobin and HGF was observed in hypertensive Japanese males and females. Since HGF is useful for evaluating hypertension-induced vascular damage, hemoglobin may be beneficial in this regard as well. Since hemoglobin is easily measured, the results indicate that it could serve as an efficient tool to evaluate vascular damage induced by hypertension in daily medical practice.

## Abbreviations

AST: aspartate aminotransferase
BMI: body mass index
GFR: glomerular filtration rate
Hb: hemoglobin
HGF: hepatocyte growth factor
SD: standard deviation
SLS: sodium lauryl surfate
β: parameter estimate
γ-GTP: γ-glutamyltranspeptidase
